# Rapid and Efficient FISH using Pre-Labeled Oligomer Probes

**DOI:** 10.1038/s41598-018-26667-z

**Published:** 2018-05-29

**Authors:** Nomar Espinosa Waminal, Remnyl Joyce Pellerin, Nam-Soo Kim, Murukarthick Jayakodi, Jee Young Park, Tae-Jin Yang, Hyun Hee Kim

**Affiliations:** 1 Chromosome Research Institute, Department of Life Science, Sahmyook University, Seoul, 01795 Korea; 2 Department of Molecular Biosciences, Kangwon National University, Chuncheon, 24341 Korea; 3 Department of Plant Science, Plant Genomics and Breeding Institute, and Research Institute of Agriculture and Life Sciences, College of Agriculture and Life Sciences, Seoul National University, Seoul, 08826 Korea

## Abstract

Fluorescence *in situ* hybridization (FISH) is used to visualize the distribution of DNA elements within a genome. Conventional methods for FISH take 1–2 days. Here, we developed a simplified, rapid FISH technique using pre-labeled oligonucleotide probes (PLOPs) and tested the procedure using 18 PLOPs from 45S and 5S rDNA, *Arabidopsis*-type telomere, and newly-identified *Panax ginseng*-specific tandem repeats. The 16 developed rDNA PLOPs can be universally applied to plants and animals. The telomere PLOPs can be utilized in most plants with *Arabidopsis*-type telomeres. The ginseng-specific PLOP can be used to distinguish *P. ginseng* from related *Panax* species. Differential labeling of PLOPs allowed us to simultaneously visualize different target loci while reducing the FISH hybridization time from ~16 h to 5 min. PLOP-FISH is efficient, reliable, and rapid, making it ideal for routine analysis, especially of newly sequenced genomes using either universal or specific targets, such as novel tandem repeats identified from whole-genome sequencing data.

## Introduction

Fluorescence *in situ* hybridization (FISH) is a powerful technique for visualizing the chromosomal location of a target DNA sequence in its native cellular environment. The technique has undergone several useful innovations since its first use in human chromosomes in 1986^1^. There are many applications for FISH, such as the detection of repetitive elements and single genes on a chromosome^2^, integration of physical and genetic maps^3^, detection of chromosomal translocations^4^, clarification of phylogenetic relationships^5^, quantification of mRNA transcripts^6^, diagnosis of hematologic cancers^7^, validation of genome assemblies^8^, and investigation of plastid dynamics^9^.

The power of FISH relies on the nature of nucleic acid probes to hybridize to complementary target sequences, the availability of several probe-labeling systems, the wide array of available fluorochromes, and refinements in fluorescence microscopy^10–12^. A well-prepared chromosome spread and an efficiently labeled probe are prerequisites for a successful FISH experiment. Several enzymatic methods for labeling probes include nick-translation, random priming, and PCR. Of these, nick-translation is used most often^11^. In nick-translation, probes are labeled with nucleotide analogs conjugated with either a hapten or a fluorochrome, which is referred to as indirect and direct labeling, respectively^11,13^. Hapten binding is visualized using a subsequently applied antibody conjugated with a fluorochrome. Additional steps and reagents are needed for indirect labeling compared with direct labeling, although these techniques have their respective advantages and disadvantages in terms of signal sensitivity and ease.

The major drawbacks of conventional FISH include the excellent technical skills needed to produce good, reproducible results^14^, the high cost of individual reagents, and the sensitivity of the enzymes used in nick translation labeling to repeated freeze-thaw cycles^15,16^. When setting up a FISH experiment, it is important to consider which approach to take based on the objectives of the experiment and the frequency with which FISH analysis will be carried out. For example, tandem repeat targets may require a slightly different probe design and FISH approach compared with unique genic targets. Moreover, readily available, specific predesigned probes are more efficient than nick-translation probes for routine FISH analysis.

In FISH karyotyping and cytotaxonomy, satellite DNAs (satDNAs) such as 5S and 45S ribosomal DNAs (rDNA) are frequently utilized as probes due to the high conservation of their coding regions among eukaryotic genomes^17,18^. The 45S rDNA and 5S rDNA sequences encode ribosomal RNAs that are needed for proper ribosome function and protein synthesis. Although not all repeat units are transcriptionally active, 45S and 5S rDNA are present in up to several thousand copies in tandem arrays at either single or multiple chromosomal loci^17,19,20^. In addition, the use of telomeric sequences has also been vital for elucidating the evolution of some plants as well as the pathophysiology of certain diseases such as cancer^21,22^. These sequences, such as TTAGGG_n_ and TTTAGGG_n_, which are found in many vertebrates and plants, respectively, are vital for chromosome integrity and are therefore generally present in chromosome termini, although recent studies have revealed several variations from these canonical repeat sequences^21^.

Conventional probe preparation involves cloning or PCR amplification and labeling of a relatively long repeat sequence, a time-consuming process^23^. The use of pre-labeled oligonucleotides as probes would make FISH analysis both cost- and time-efficient. In this study, we developed a FISH analysis method by designing pre-labeled oligonucleotide probes (PLOP) and a highly efficient reproducible hybridization protocol (PLOP-FISH). We demonstrate a variety of applications for PLOP-FISH for examining universal targets, as well as species-specific high copy number tandem-repeat blocks that were newly identified from next-generation sequencing (NGS) platform-WGS data.

## Results

### Designing universal 45S rDNA, 5S rDNA, and telomere PLOPs

**Figure 1:**
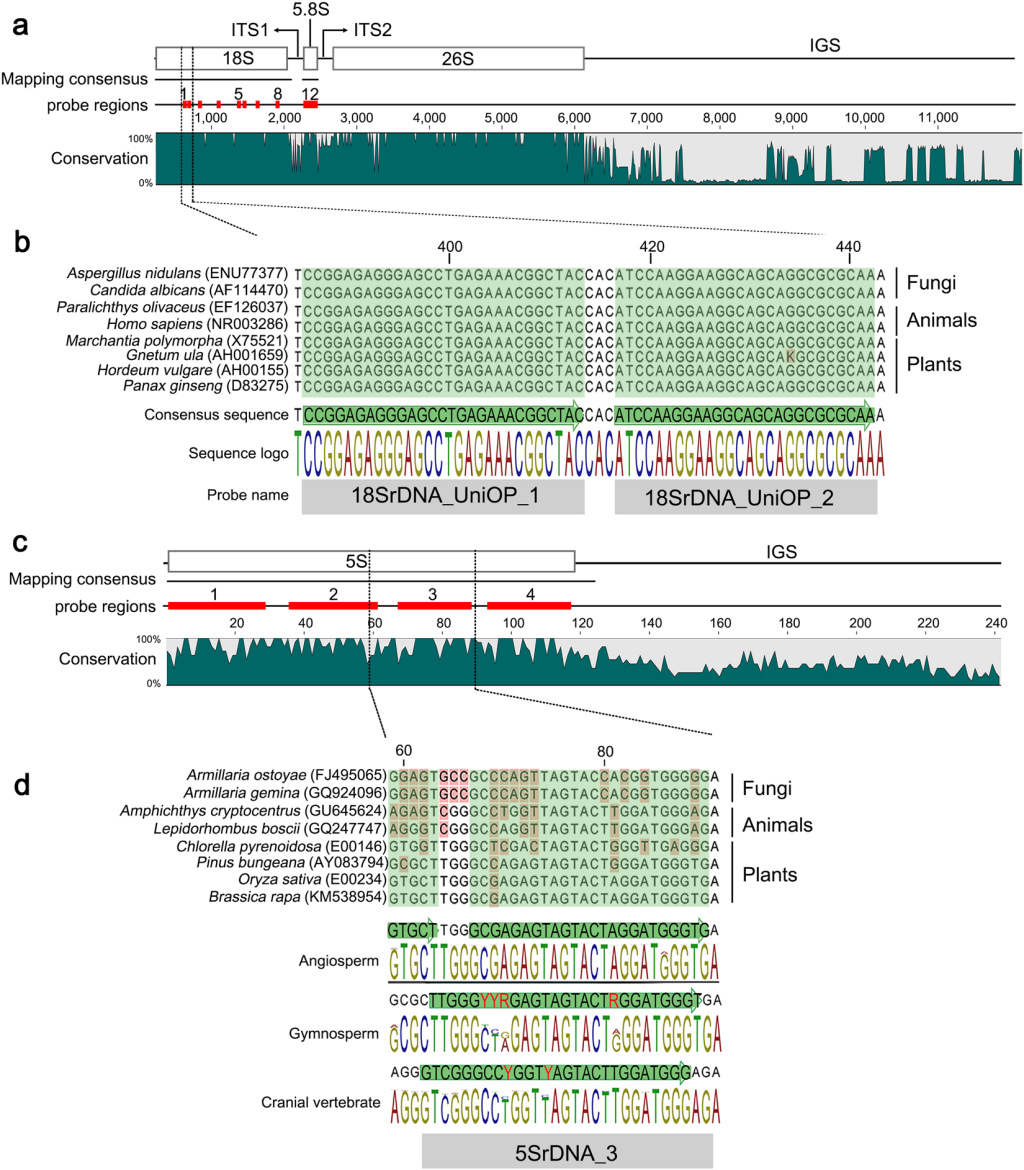
Design of the 45S and 5S rDNA PLOPs. (**a**) Diagram of the 45S rDNA unit of *P. ginseng* (KM036296), as commonly observed in most plant species. The consensus region was generated from mapping sequences downloaded from public databases (Supplementary Fig. S1). The 12 PLOP regions for 45S rDNA are indicated by red bars. The sequence conservation for each region of the 45S rDNA is represented by homologous sequence depth across 32 complete rDNA-IGS from different species. **(b)** Two examples of PLOPs in the 18S region of 45S rDNA. All sequences are conserved across different fungal, animal, and plant taxa. The bottom part of the figure shows the consensus sequence, sequence logo, and probe names. **(c)** Diagram of the 5S rDNA unit of *P. ginseng* (KM036312), as commonly observed in most plant species. The four 5S rDNA PLOP regions are indicated by red bars, and the sequence conservation is represented as the depth of homologous sequences across eight species. **(d)** An example of PLOPs based on 5S rDNA. The 5S rDNA coding regions are highly polymorphic across distantly related taxa. To address this issue, different PLOPs were designed for each lineage of angiosperm, gymnosperm, and cranial vertebrate species (see Table 1). The consensus sequence, sequence logo, and probe name are shown. Degenerate nucleotides (red nucleotides in the consensus sequences) were incorporated into the probes (thick green arrows) to include polymorphic regions, and each type was pooled to form the “universal” 5S rDNA FISH probe cocktail.

We identified 414, 7,249, and 6,750 sequences for 18S, 5.8S, and 5S rDNAs, respectively, from public databases. We aligned the 18S and 5.8S rDNA sequences and the 5S rDNA sequences to the complete *Panax ginseng* 45S rDNA (KM036296) and 5S rDNA (KM036312) units as references, respectively. Of these, 76%, 66%, and 60% of the 18S, 5.8S, and 5S rDNA sequences, respectively, were mapped to the reference sequences (Supplementary Fig. S1a,b, Supplementary Table S1). The reference mapping generated a 1,851-bp, 201-bp, and 124-bp consensus sequences for 18S, 5.8S and 5S rDNA, respectively, with reads derived from fungi, animals, and plants (Fig. 1, Supplementary Fig. S1, and Supplementary Table S2). The species and accession numbers of the sequences that were mapped to the reference sequences are listed in Supplementary Tables S3–S5.

The PLOPs were designed based on the most highly conserved regions in 45S and 5S rDNA repeats based on sequence mapping and alignment. For the 45S rDNA repeat, the 18S and 5.8S rRNA genes showed conserved sequence homology among fungi, animal, and plants. Twelve universal PLOPs were designed based on the conserved regions of 45S rDNA genes, including eight from 18S rRNA and four from 5.8S rRNA. Four PLOPs were designed based on 5S rDNA (Fig. 1 and Table 1). All oligomers are 25–31 bp long, with melting temperatures (Tm) of 39 to 53 °C at 2 × SSC and 50% formamide (Fig. 1, Supplementary Fig. S1, and Table 1).

**Table 1:** List of oligoprobes used in this study.

No.	Probe	Sequence	Length	Position in consensus	Tm (°C)^*^	Modification
1	18SrDNA_UniOP_1	CCGGAGAGGGAGCCTGAGAAACGGCTAC	28	386…413	49.2	5′-Cy3
2	18SrDNA_UniOP_2	ATCCAAGGAAGGCAGCAGGCGCGCAA	26	417…442	50.9	5′-Cy3
3	18SrDNA_UniOP_3	GGGCAAGTCTGGTGCCAGCAGCCGCGGT	28	555…582	53.4	5′-Cy3
4	18SrDNA_UniOP_4	TCGAAGACGATYAGATACCGTCSTAGT	27	994…1020	40.4–43.4	5′-Cy3
5	18SrDNA_UniOP_5	CTGAAACTTAAAGGAATTGACGGAAGG	27	1133…1159	40	5′-Cy3
6	18SrDNA_UniOP_6	GGAGCCTGCGGCTTAATTTGACTCAAC	27	1174…1200	45.0	5′-Cy3
7	18SrDNA_UniOP_7	GGTGGTGCATGGCCGTTCTTAGTTGGTGG	29	1272…1300	47.1	5′-Cy3
8	18SrDNA_UniOP_8	ACGTCCCTGCCCTTTGTACACACCGCCCGTC	31	1622…1652	51.0	5′-Cy3
9	5.8SrDNA_UniOP_1	AAYGACTCTCGGCAACGGATATCTMG	26	16…41	42.0–44.9	5′-Cy3
10	5.8SrDNA_UniOP_2	CWYGCATCGATGAAGAACGTAGCRA	25	45…69	42.2–45.2	5′-Cy3
11	5.8SrDNA_UniOP_3	GCGATACTTGGTGTGAATTGCAGAATC	27	73…99	42.0	5′-Cy3
12	5.8SrDNA_UniOP_4	GTGAACCATCGAGTYTTTGAACGCAAGT	28	102…129	43.4	5′-Cy3
13	5SrDNA_ang_1	GGATGCGATCATACCAGCACTAAAGCACCG	30	1…30	48.2	5′-Alexa Fluor 488
14	5SrDNA_gym_1	GRGTGCGATMATACCASCGYTWRYGYA	27	1…27	38.4–48.0	5′-Alexa Fluor 488
15	5SrDNA_cranial_1	GYYTAYRGCCAYACCACCCTGRRHRCG	27	1…27	37.8–48.3	5′-Alexa Fluor 488
16	5SrDNA_ang_2	CCCATCAGAACTCCGAAGTTAAGCGTGCT	29	34…62	47.4	5′-Alexa Fluor 488
17	5SrDNA_gym_2	ATCCSATCAGAACTCCGYARTTAAGCR	27	32…58	39.7–45.6	5′-Alexa Fluor 488
18	5SrDNA_cranial_2	GATCTCGTCYGATCTCGGAAGCTAAGC	27	31…57	45.6–46.8	5′-Alexa Fluor 488
19	5SrDNA_ang_3	GCGAGAGTAGTACTAGGATGGGTG	24	66…89	40.7	5′-Alexa Fluor 488
20	5SrDNA_gym_3	TTGGGYYRGAGTAGTACTRGGATGGGT	27	62…88	38.4–42.2	5′-Alexa Fluor 488
21	5SrDNA_cranial_3	GTCGGGCCYGGTYAGTACTTGGATGGG	27	61…87	44.4–46.6	5′-Alexa Fluor 488
22	5SrDNA_ang_4	CCTGGGAAGTMCTCGTGTTGCAYYCC	26	94…119	40.7–42.2	5′-Alexa Fluor 488
23	5SrDNA_gym_4	CTCYYGGGAAGTCCYRRTRTYGCACCC	27	92…118	41.9–44.8	5′-Alexa Fluor 488
24	5SrDNA_cranial_4	CYGCCTGGGAATACCRGGTGYYGTARG	27	91…117	41.9–48.6	5′-Alexa Fluor 488
25	Tel_UniOP_*Arabidopsis*	TTTAGGGTTTAGGGTTTAGGGTTTAGGGT	29	na	40.27	ATTO425
26	Pgms1	ACATTCTTGATACATTCTTGATACATTCTT	30	na	38.13	5′-Cy3

^*^At 2 × SSC and 50% Formamide.

The 5S rDNA coding sequences show relatively diverse sequence similarities across distantly related taxa (Fig. 1c). The initial 5S rDNA FISH results for *Ginkgo biloba* using 5S rDNA PLOPs designed from angiosperm sequences showed weak hybridization. Signals were detected only in loose interphase chromatin and not in condensed metaphase chromosomes, even after several FISH attempts (Supplementary Fig. S2). A similar pattern was observed in the flounder fish *Paralichthys olivaceus* (Supplementary Fig. S3). These results indicate that the angiosperm-derived 5S rDNA PLOPs hybridize poorly to the highly packed *G. biloba* and *P. olivaceus* metaphase chromosomes due to sequence divergence of the angiosperm-derived PLOPs compared to those of gymnosperm and vertebrate 5S rDNA target sequences. To overcome this issue and to detect 5S rDNA loci in as many distantly related taxa as possible, we designed additional 5S rDNA PLOPs based on gymnosperm and cranial vertebrate sequences. To obtain a more comprehensive representation of 5S rDNA sequences from divergent taxa, we carried out independent multiple alignments for angiosperms, gymnosperms, and cranial vertebrates and designed PLOPs with manually substituted degenerate nucleotides for each group to include all possible sequences (Fig. 1d, Supplementary Table S2).

The 45S rDNA probe cocktail consisted of 12 PLOPs labeled with Cy3 at the 5′ end, whereas for 5S rDNA, four PLOPs labeled with FAM at the 5′ end were pooled to produce an independent 5S rDNA probe cocktail for angiosperms, gymnosperms, and cranial vertebrates. In addition to the rDNA PLOPs, we designed an *Arabidopsis*-type telomeric probe to produce a probe for plant telomeres (Table 1).

### Optimization of PLOP-FISH

We initially tested the hybridization efficiency of the PLOP cocktails by measuring the signal intensity of each repeat in *Zea mays* metaphase chromosomes at different durations of hybridization. Distinct, reproducible signals for 45S rDNA, 5S rDNA, and *Arabidopsis*-type telomere were observed at 5 min, 1 hr, and 7 hrs (Supplementary Fig. S4a–c). Since the hybridization time did not affect the FISH signals, for the remaining experiments, hybridization was carried out for 1 hr. In addition, we evaluated the signal intensity of the PLOPs in interphase cells. Accordingly, distinct signals were observed in *Z. mays* interphase cells (Supplementary Fig. S4d). Altogether, these results indicate that the PLOPs hybridize efficiently to *Z. mays* target sequences at both the loose and condensed chromatin stages.

### Universal rDNA PLOPs for diverse taxa

**Figure 2:**
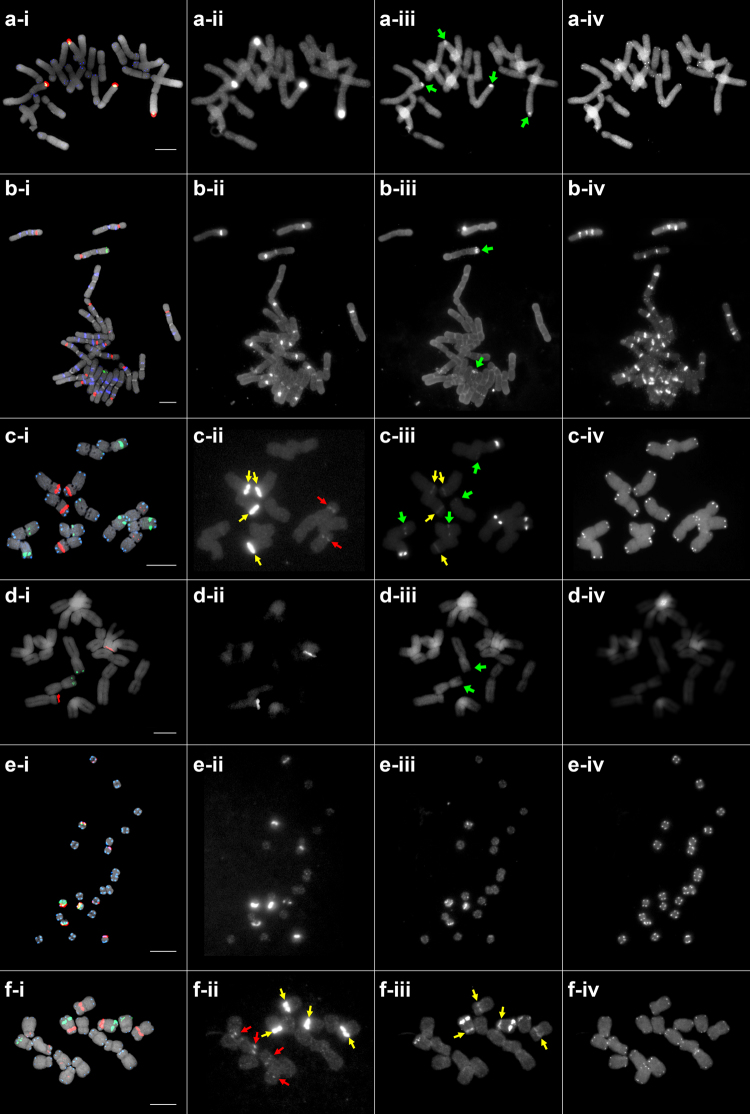
PLOP-FISH signals in gymnosperm and angiosperm species. (**a**) *Ginkgo biloba* and (**b**) *Pinus densiflora*, (**c**) *Hordeum vulgare*, (**d**) *Allium cepa*, (**e**) *Phaseolus vulgaris*, (**f**) *Trigonella foenum-graecum*. Panels i–iv show the merged, raw 45S rDNA, raw 5S rDNA, and raw telomeric PLOP signals, respectively. Red, green, and blue signals in a-i – f-i represent 45S rDNA, 5S rDNA, and telomere, respectively. Red, green, and yellow arrows indicate weak 45S, weak 5S, and 45S signal crosstalk in the FITC filter, respectively. Bars = 10 μm.

We evaluated the versatility of the “universal” PLOP-FISH cocktail by performing FISH analysis on ten angiosperm, two gymnosperm, and one animal species (Supplementary Table S6). The 45S rDNA signals showed efficient hybridization to all plant and animal species included in this study (Fig. 2, and Supplementary Figs S3 and S5–S6), indicating the universal utility of the 45S rDNA PLOPs. FISH analysis of the pooled angiosperm, gymnosperm, and cranial vertebrate-derived 5S rDNA PLOPs also allowed easy detection of 5S rDNA loci in interphase and metaphase chromosomes in all species investigated (Fig. 2 and Supplementary Figs S4–S6).

In addition, the background signal was low for both PLOPs in all chromosome spreads across all species, indicating that hybridization of the PLOPs was highly specific and that non-target signals were efficiently washed off. The very high signal-to-background ratio enabled us to detect very weak signals that could not easily be distinguished using conventional FISH methods. For example, several very weak 45S rDNA signals that were not previously observed were detected in *Hordeum vulgare* and *Trigonella foenum-graecum* (Fig. 2c,f)^24,25^. This approach allowed us to readily detect rDNA loci, revealing that most angiosperm species in this study had fewer than ten 45S rDNA loci, regardless of chromosome number. The gymnosperm *Pinus densiflora* had the highest number of 45S rDNA loci (14 loci, Fig. 2b), with the lowest chromosome number-to-45S rDNA loci ratio and the highest 45S-to-5S rDNA ratio (Supplementary Table S6). Except for *Triticum aestivum*, *H. vulgare*, and x*Triticosecale*, all species had equal numbers of 5S rDNA and 45S rDNA sequences or fewer 5S rDNA sequences.

### Multiplex FISH using PLOPs for rDNA and telomere detection

Modifying the oligonucleotides from different repeat families using fluorochromes with different excitation and emission wavelengths allowed us to perform multiplex FISH analysis. Using Cy3, Alexa Fluor 488, and ATTO425 to modify the 45S rDNA, 5S rDNA, and *Arabidopsis*-type telomere PLOPs, red, green, and blue signals were easily detected when using the appropriate narrow-band pass filters to select the specific emission wavelengths for each fluorochrome.

Multiplex PLOP-FISH analysis represents a marked improvement over the standard FISH procedure. This technique produced high-resolution signals and allowed us to readily detect colocalized rDNA signals using the same chromosome spread. For example, whereas 5S and 45S rDNA sequences in most angiosperm species are often localized in separate chromosomal regions, a few reports described the physical linkage of the 5S rDNA unit in the IGS region with the 45S rDNA unit in some Asteraceae species and in *G. biloba*^26^. Indeed, all 5S and 45S rDNAs were detected in the same chromosomal regions in *G. biloba* (Fig. 2a). Additionally, seemingly colocalized 5S and 45S rDNA signals were observed in *H. vulgare*, *T. foenum-graecum* (Fig. 2c,f), *Z. mays* (Fig. S4), *T. aestivum* and x*Triticosecale* (Fig. S5b,c) when we used 12 PLOPs for 45S rDNA. However, FISH using four of the 12 45S rDNA PLOPs (Table 1 nos. 1~4) and four angiosperm 5S rDNA (Table 1 nos. 13, 16, 19, 22) produced no colocalization in *H. vulgare* and *T. foenum-graecum* (Fig. S7). This indicates a false 45S and 5S colocalization in these species but rather a mere signal crosstalk of the Cy3 fluorescence in the FITC filter, likely caused by over brightness of the 45S rDNA loci.

While we detected FISH signals in most species using the *Arabidopsis*-type telomere PLOP, there were some exceptions. The gymnosperms *G. biloba* and *P. densiflora*, which are phylogenetically distant from *Arabidopsis*, showed signals using the *Arabidopsis*-type telomere probe, while the monocot angiosperm *Allium cepa* did not show any hybridization, supporting the fact that the telomere repeat in this genus is divergent from that of the plant consensus sequence TTTAGGG found in *A. thaliana*^27^. As expected, sequences from the animal species *P. olivaceus* failed to hybridize to the plant-derived *Arabidopsis*-type telomere PLOP (Supplementary Fig. S3).

### PLOP-FISH to detect a novel *Panax ginseng*-specific repeat

Universal PLOP-FISH analysis of rDNA sequences makes it easy to perform preliminary karyotyping, particularly for species without prior karyotype data. However, the use of rDNA signals often allows only a limited number of homologous chromosomes to be identified. Additionally, the distribution of rDNA signals may not vary in closely related species and may therefore not provide additional evolutionary information. Thus, identifying satDNAs unique to a certain group of taxa or species would be ideal for efficient karyotyping and genome evolutionary studies. Moreover, the rapid, high-throughput identification of specific repeats can be performed using WGS data produced on an NGS platform.

One highly efficient method for identifying repeats from WGS reads is the Galaxy-based RepeatExplorer pipeline^28^. Using the Tandem Repeat Analyzer (TAREAN)^29^ workflow of RepeatExplorer, we identified several repeats in *Panax ginseng*, including Pg167TR, a high-copy number satDNA that has been used to refine the *P. ginseng* karyotype^30^. Another newly identified repeat is the 11-bp (ACATTCTTGAT) minisatellite, Pgms1. We investigated the redundancy of Pgms1 by mapping WGS reads derived from four *Panax* species, including two diploids (*P. notoginseng* and *P. vietnamensis*) and two tetraploids (*P. ginseng* and *P. quinquefolius*). WGS reads mapping revealed that Pgms1 is specific to *P. ginseng* and is not found in the other species examined (Supplementary Fig. S8a). This result is supported by FISH data (Supplementary Fig. S8b–d). Pgms1 is localized in the short arm of *P. ginseng* chromosome 1 (Supplementary Fig. S8b), and it was not observed in *P. notoginseng* or *P. quinquefolius* (Supplementary Fig. S8c,d). In addition, we identified telomeric repeats using the same approach^31^; FISH revealed that all of these sequences are localized to all *P. ginseng* chromosome termini, with an additional interstitial site detected on one chromosome (Supplementary Fig. S8e).

## Discussion

Pre-labeled oligomers have been widely utilized to detect specific bacterial strains and human cell lines in microbial and human clinical studies by exploiting polymorphic DNA or RNA sequences^32,33^. This approach takes advantage of conserved and lineage-specific polymorphisms in ribosomal RNA genes (rDNA)^34^. By exploiting these features of rDNA, probes can be designed with varying specificity to identify different taxonomic groups at the species, genera, or even domain level^33^. In plants, PLOPs have been utilized to localize chromosome- or genome-specific repeats and unique sequences^35,36^. They have also been used to replace cumbersome BAC probe preparation in wheat and rye^37^. However, although the use of pre-labeled probes has been popular in microbiological and some plant studies, these probes have not been widely exploited as universal rDNA probes. Here, we bioinformatically designed PLOPs and performed FISH using an integrated method from previously published methods^32,33,36–38^

In plant cytogenetics, rDNAs are commonly utilized for preliminary FISH karyotyping for species without prior karyotype data. When this analysis is routinely conducted with different species, the labeling of cloned or PCR-amplified rDNA probes with haptens or fluorochromes through nick-translation is often costly and time-consuming and can yield inconsistent results^15,23^. Reduced enzyme activity after the inevitable repeated freeze-thaw cycles is one cause of batch-associated labeling inconsistencies. Here, we developed alternative approach using PLOPs designed from highly conserved regions of rDNAs that can hybridize to target sequences of distantly related taxa, unlike the species-specific approach used in most microbiology studies. We further pooled these probes to serve as a “universal” rDNA FISH probe cocktail for routine analysis.

Our results demonstrate the potential of this approach for use at a diverse taxonomic level. Although we analyzed only a few species in this study, more species including fungi should be analyzed to further validate these PLOPs in future studies. However, we expect that reproducible signals will be obtained in other species, since these probes were designed based on highly conserved regions in plants, animals, and fungi, and all species included in this study showed excellent signals. However, it is important to use high-quality slides with good chromosome spreads for optimal hybridization and signal detection.

The *Arabidopsis*-type telomere repeat TTTAGGG is considered to be a consensus sequence in higher plants^39^. Accordingly, most plant species in this study generated telomere signals using the *Arabidopsis*-type PLOP, albeit with varying signal intensities, except for *A. cepa*, which showed no signals (Fig. 2, Supplementary Figs S5–S6). Several plant species, such as those in the order Asparagales, have telomere sequences that diverged from the *Arabidopsis*-type sequence, instead consisting primarily of the vertebrate-type TTAGGG sequence^40^. In addition, a few genera in the Solanaceae family carry the TTTTTTAGGG sequence^40^, and telomere sequences of the genus *Allium* were recently shown to have diverged from the consensus sequence to CTCGGTTATGGG^21^, which explains why no *Arabidopsis*-type telomere repeat signal was observed in *A. cepa* (Fig. 2d).

The rDNA FISH signals are consistent with a previous report of colocalization for the 5S and 45S rDNA loci in *G. biloba*^41^. The 5S and 45S rDNA clusters are linked together as one repeat unit (i.e., 5S rDNA is inserted in the IGS region of the 45S unit), which likely occurred during the early evolution of plants. This type of cluster is known as the L-type, in contrast to the separate (S)-type commonly observed in most higher plants^26^. The separation of the two rDNA repeat families is thought to have occurred in the early land plants, whereas the reintegration of 5S rDNA into the 45S rDNA IGS has been reported in some gymnosperm and angiosperm species^26,41–43^. Extensive molecular analyses have been performed in species within the gymnosperm families Ephedraceae, Ginkgoaceae, Podocarpaceae and the angiosperm genus *Artemisia* in the family Asteraceae, revealing the existence of either exclusive L-type rDNA organization or coexisting L-type and S-types^26,41–43^. However, more extensive molecular cytogenetic analysis across all plant species is necessary to get an even better understanding of the evolutionary dynamics of the two rDNA families, especially because the number of plant species with molecular cytogenetic data is still very low (<2,000) compared to the number of seed plants in the world (>400,000)^44,45^.

**Figure 3:**
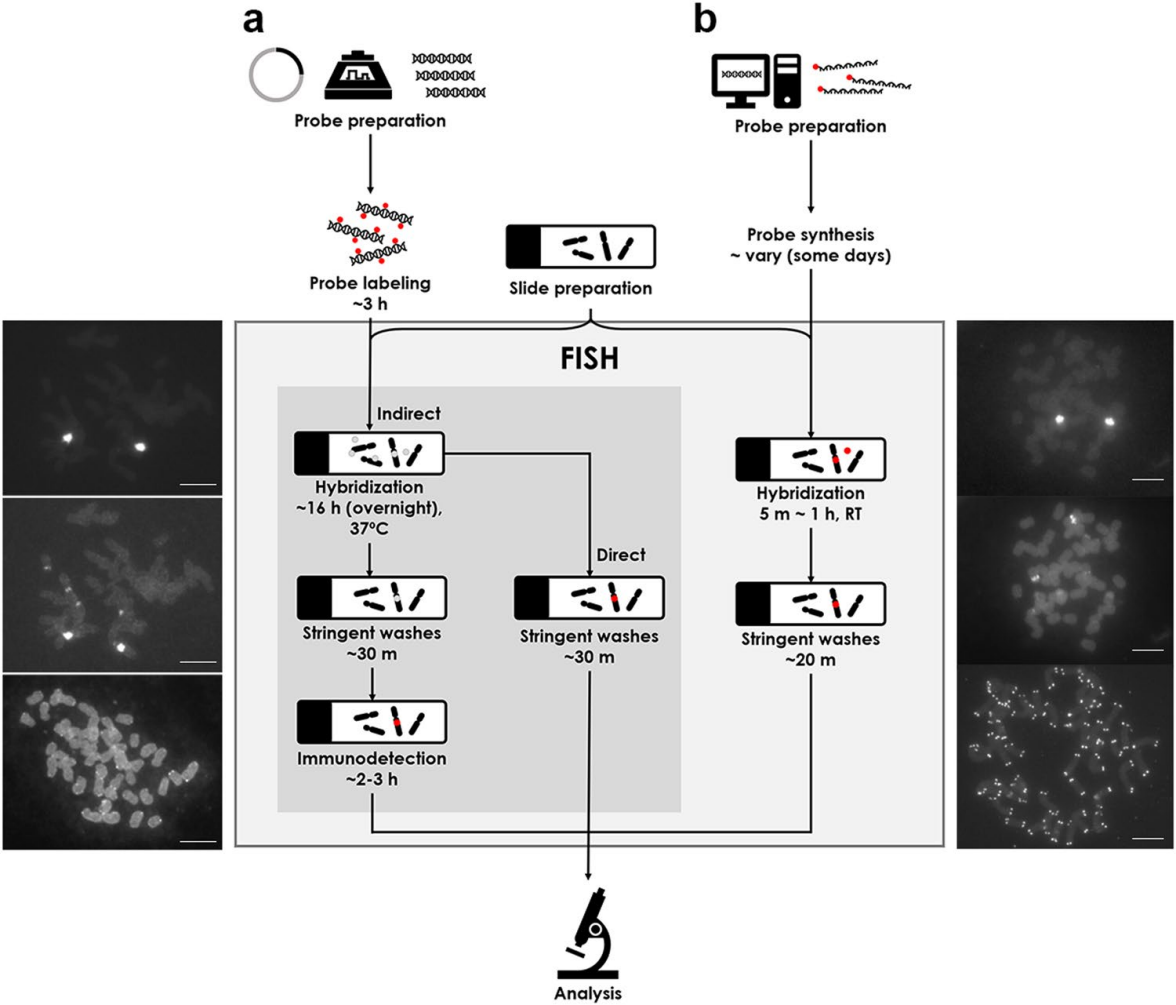
Schematic diagram comparing the conventional FISH and PLOP-FISH workflows. (**a**) Probe preparation prior to FISH may involve cloning or PCR amplification of a target sequence and subsequent nick-translation labeling with either haptens (indirect) or fluorochromes (direct), producing ~200–500 bp probes. FISH using these relatively long double-stranded DNA probes involves overnight hybridization at 37 °C, and if the probe is labeled with a hapten, immunodetection should be carried out. Conventional FISH using indirectly labeled probes by nick-translation typically involves additional steps such as RNase and protease treatment prior to the hybridization reaction (not shown in diagram) and an immunodetection step. Probes directly labeled with fluorochromes may or may not be treated with RNase and protease. Both methods need overnight hybridization, making them more time consuming and labor intensive than PLOP-FISH. (**b**) PLOP-FISH begins with the design of probes from bioinformatically analyzed sequences to optimize probe length (~30 bp) and melting temperature (~45–50 °C at 2 × SSC and 50% formamide) for rapid hybridization at room temperature. Ordering of fluorochrome-prelabeled oligonucleotide sequences eliminates the labeling step but may take some days to be delivered. Probe preparation for both conventional and PLOP-FISH may vary depending on the nature of probe source. The general processes and number of steps required for PLOP-FISH are reduced compared with conventional FISH, thus reducing the chances for error, while simplifying and expediting downstream analyses. Inset images in the left and right panels show conventional and PLOP-FISH signals of 45S rDNA, 5S rDNA, and telomere (top to bottom), respectively. Bars = 10 µm.

The FISH results presented here demonstrate the robustness and reproducibility of the PLOP-FISH technique. For example, a minor 45S rDNA signal that had not previously been detected in the monocot *Hordeum vulgare* or the dicot *T. foenum-graecum* was observed using the PLOPs developed in this study (Figs 2c,f and 3)^24,25^. However, prudence is needed when using all 12 of the 45S rDNA PLOPs as they could also produce signal crosstalk with other filters. It is, therefore, important to either reduce the number of PLOPs of very intense targets (i.e. 45S rDNA) and avoid overexposure of fluorescence.

This method can be used to analyze the evolutionary relationships of related taxa using unique or common satDNAs. Furthermore, probes designed from InDel regions of rDNA repeats can be used to confirm genetic and evolutionary diversity through chromosomal visualization of sequence variants, as was done in several *Allium* species^33,34,46^. This approach can also be performed using other types of tandem repeats, such as micro- and minisatellites common to a specific group of species, for use as cytogenetic markers in comparative evolutionary studies, such as Pgms1 from *P. ginseng*.

When performing routine high-throughput analysis, time is a crucial factor; more data obtained in a shorter timespan means higher productivity. The method presented here allows high-quality data to be acquired using a hybridization time as short as 5 min instead of the usual 16-h incubation time required using conventional nick-translation-derived rDNA probes. In addition, no incubator is required since hybridization is performed at room temperature. However, it is important to consider the melting temperature when designing PLOPs, as this factor influences the hybridization efficiency at room temperature. Our experiments with PLOPs produced clean and distinct signals even with shorter stringent washes compared with nick-translation-prepared probes, most likely due to the much shorter probe length than those derived from nick translation (typically 200–500 bp).

In terms of reagent cost, a 100 μl volume of pre-labeled oligomer at a concentration of 100 pmol/μl could be utilized for more than 3,000 slides when using 40 µl of FISH mixture. This could be maximized to 12,000 slides when using only 10 µl total volume of hybridization mixture. The oligoprobe price will vary depending on the length, synthesis scale, purification method, fluorophore, and number of modifications (i.e. either only 5′ or 3′ or both). For a 50-nmole 30-bp oligonucleotide labeled with Cy3 at the 5′ end and purified through HPLC, the probe costs about 180 USD. To put this price into perspective, a 40-µl and 10-μl FISH experiment would cost about 0.06 and 0.01 USD/slide, compared to 2.16 and 0.54 USD for the direct-labeling method. Therefore, the current technique represents a highly simplified, rapid, reproducible, and cost-efficient FISH process (Fig. 3).

**Figure 4:**
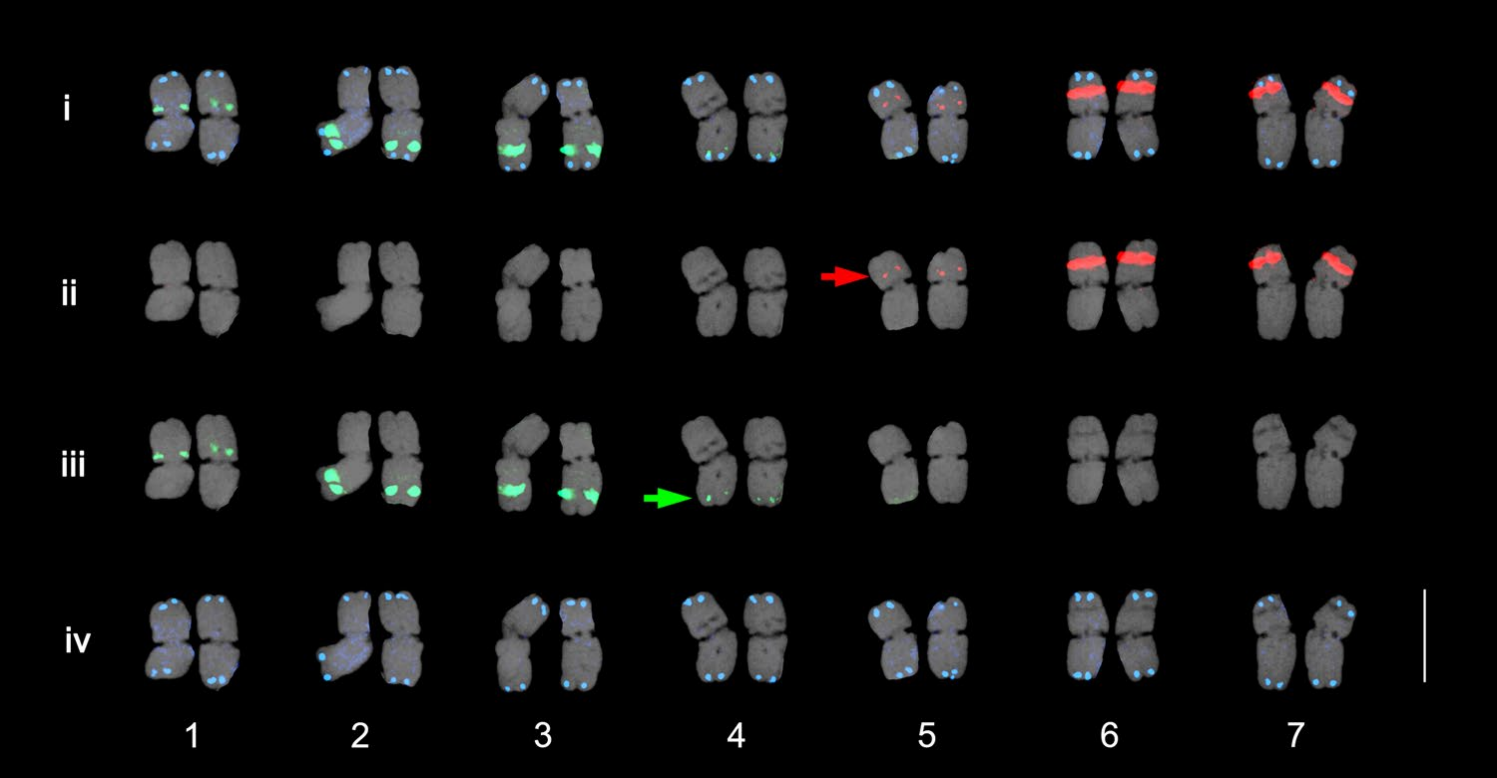
An application of PLOP-FISH for rapid and efficient karyotyping in *Hordeum vulgare*. Using the 45S rDNA (red signals), 5S (green signals), and *Arabidopsis*-type telomere (blue signals) PLOP cocktail, all seven homologous chromosomes were easily identified. Panels i-iv show merged, 45S rDNA, 5S rDNA, and telomere signals, respectively. The karyogram shows weak 45S rDNA signals (red arrow) not detected in Brown, *et al*.^24^. The green arrow indicates weak 5S rDNA signals. Bar = 10 μm.

This rapid PLOP-FISH method can expedite routine karyotyping analysis using tandemly repeated sequences (Fig. 4). The mining of abundant repetitive elements can be carried out using a *de novo* repeat analyzer such as the RepeatExplorer or TAREAN. This process does not require the use of scaffold assemblies, instead requiring only low-coverage WGS reads^28,29^, thereby accelerating FISH analysis for high-resolution visualization of complex chromosomes, even for genomes without prior genome assembly data.

## Methods

### PLOP Design

Eukaryote 5S, 5.8S, and 18S rDNA sequences were obtained from the NCBI nucleotide database (https://www.ncbi.nlm.nih.gov/). Additional 5S rDNA sequences were obtained from the 5S rDNA database (http://combio.pl/rrna/)^47^. To identify conserved regions for each rDNA family across a wide range of species, individual downloaded sequences from each rDNA family were treated as a single read, and the sequences were mapped if a minimum of 50 nt matched the reference sequence using a medium stringency parameter in CLC Main Workbench Version 7.8.1 software (CLC Inc., Rarhus, Denmark). The 5S rDNA sequences were mapped to the *Panax ginseng* 5S rDNA reference sequence (KM036312), and the 5.8S and 18S rDNA sequences were mapped to the *Panax ginseng* 45S rDNA reference sequence (KM036296), using CLC Main Workbench. Consensus sequences were generated through mapping, and probes were designed based on the consensus sequences. Twelve 24–31 bp PLOPs spanning the 18S and 5.8S consensus sequences were designed, whereas four were designed for 5S rDNA (Fig. 1 and Supplementary Fig. S1). The 5.8S and 18S rDNA PLOPs were pooled to detect the 45S rDNA site. In addition to rDNA probes, a probe was designed based on the *Arabidopsis*-type telomeric DNA sequence (TTTAGGG)_4_. The PLOPs were 5′-labeled with Alexa Fluor 488 (5S rDNA), Cy3 (5.8S and 18S), and Atto425 (*Arabidopsis*-type telomere) through chemical method as provided by Bioneer Corporation (South Korea).

### Plant and animal samples used for FISH analysis

The institutions listed in Supplementary Table S6 provided the plant and animal materials used to validate the efficiency of the PLOPs across various distantly related taxa. Fixed gonads of *P. olivaceus* were received from Dr. Woo Jin Kim in National Institute of Fisheries Science, Pusan 46083, Republic of Korea.

### FISH analysis

Mitotic metaphase chromosome spreads were produced following a previous method^30^. Thirty-two microliters of FISH hybridization master mix (50% formamide, 10% dextran sulfate, and 2 × SSC) and 25 ng of each PLOP (5S and 45S rDNAs and *Arabidopsis*-type telomeric repeats) were combined, followed by the addition of distilled water to a total volume of 40 µl. Chromosomal DNA on a glass slide was denatured at 80 °C for 5 min after the addition of hybridization mix. To determine the most rapid FISH procedure that did not compromise FISH signal quality, hybridization durations of 5 min, 1 h, and 7 h at room temperature were evaluated. Stringency washes were performed using 2 × SSC at room temperature for 5 min, 0.1 × SSC at 42 °C for 10 min, and 2 × SSC for 5 min at room temperature. The slides were dehydrated in an ethanol series of 70%, 90%, and 100%, air-dried, and counterstained with premixed 4′,6-diamidino-2-phenylindole (DAPI) solution (1 μg/ml; DAPI in Vectashield, Vector Laboratories, Burlingame, CA, USA). Images were captured under a model BX53 fluorescence microscope (Olympus, Tokyo, Japan) equipped with a DFC365 FS CCD camera (Leica Microsystems, Wetzlar, Germany) and processed using Cytovision ver. 7.2 (Leica Microsystems). Further image enhancements and karyogram construction were performed with Adobe Photoshop CC (Adobe Systems, San Jose, CA, USA).

### Repeat identification and WGS read mapping

Approximately 0.014× of ginseng WGS reads were used to analyze the repeats in ginseng using TAREAN^29^. Randomly extracted WGS reads from four *Panax* species (Supplementary Table S7) were mapped to a concatenated 11-bp Pgms1 using CLC Assembly Cell ver. 4.21 (http://www.clcbio.com/products/clc-assembly-cell/), using default parameters.

### Data availability

The *P. ginseng* sequencing data have been deposited at the National Agricultural Biotechnology Information Center (NABIC) with the accession code NN-0076-000001 (https://nabic.rda.go.kr:2360/ostd/basic/ngsSraView.do)^48^.

## Electronic supplementary material

Supplementary Information

Dataset 1

## References

[1] Pinkel D, Straume T, Gray J (1986). Cytogenetic analysis using quantitative, high-sensitivity, fluorescence hybridization. PNAS.

[2] Khrustaleva LI, Kik C (2001). Localization of single-copy T-DNA insertion in transgenic shallots (*Allium cepa*) by using ultra-sensitive FISH with tyramide signal amplification. Plant J..

[3] Szinay D (2008). High-resolution chromosome mapping of BACs using multi-colour FISH and pooled-BAC FISH as a backbone for sequencing tomato chromosome 6. Plant J..

[4] Huang S (2009). The genome of the cucumber, *Cucumis sativus* L. Nature Genet..

[5] Siljak-Yakovlev S, Pustahija F, Vicic V, Robin O (2014). Molecular cytogenetics (FISH and fluorochrome banding): resolving species relationships and genome organization. Methods Mol. Biol..

[6] Trcek T (2012). Single-mRNA counting using fluorescent *in situ* hybridization in budding yeast. Nat. Protoc..

[7] Hu L (2014). Fluorescence *in situ* hybridization (FISH): an increasingly demanded tool for biomarker research and personalized medicine. Biomark. Res..

[8] Chamala S (2013). Assembly and validation of the genome of the nonmodel basal angiosperm *Amborella*. Science.

[9] Martis MM (2012). Selfish supernumerary chromosome reveals its origin as a mosaic of host genome and organellar sequences. PNAS.

[10] Sommerauer, M. & Feuerbacher, I. In *Fluorescence in situ hybridization (FISH)—application guide* (ed. T. Liehr) 85-97 (Springer, Berlin, Heidelberg, 2009).

[11] Morrison, L. E., Ramakrishnan, R., Ruffalo, T. M. & Wilber, K. A. In *Molecular Cytogenetics: Protocols and Applications* (ed. Yao-Shan Fan) 21-40 (Humana Press, 2003).

[12] Liehr, T. In *Quality Issues in Clinical Genetic Services* (eds Ulf Kristoffersson, Jörg Schmidtke, & J. J. Cassiman) 315-320 (Springer Netherlands, 2010).

[13] Liehr, T. & Pellestor, F. in *Fluorescence In Situ Hybridization (FISH)—Application Guide* 23–34 (2009).

[14] Gozzetti A, Le Beau MM (2000). Fluorescence *in situ* hybridization: uses and limitations. Semin. Hematol..

[15] Kato A, Albert PS, Vega JM, Birchler JA (2006). Sensitive fluorescence *in situ* hybridization signal detection in maize using directly labeled probes produced by high concentration DNA polymerase nick translation. Biotech. Histochem..

[16] Jensen E (2014). Technical Review: *In Situ* Hybridization. Anat. Rec..

[17] Martins, C. & Wasko, A. P. In *Focus in genome research* (ed. C. R. Williams) Ch. 10, 335-363 (Nova Science Publishers, Inc., 2004).

[18] Soltis PS (1999). The phylogeny of land plants inferred from 18S rDNA sequences: pushing the limits of rDNA signal?. Mol. Biol. Evol..

[19] O’Sullivan JM, Pai DA, Cridge AG, Engelke DR, Ganley ARD (2013). The nucleolus: a raft adrift in the nuclear sea or the keystone in nuclear structure?. Biomol. Concepts..

[20] Long EO, Dawid IB (1980). Repeated genes in eukaryotes. Annu. Rev. Biochem..

[21] Fajkus P (2016). *Allium* telomeres unmasked: the unusual telomeric sequence (CTCGGTTATGGG)_n_ is synthesized by telomerase. Plant J..

[22] Epel ES (2004). Accelerated telomere shortening in response to life stress. PNAS.

[23] Silahtaroglu A, Pfundheller H, Koshkin A, Tommerup N, Kauppinen S (2004). LNA-modified oligonucleotides are highly efficient as FISH probes. Cytogenet. Genome. Res..

[24] Brown SE, Stephens JL, Lapitan NL, Knudson DL (1999). FISH landmarks for barley chromosomes (*Hordeum vulgare* L.). Genome.

[25] Ahmad F, Acharya SN, Mir Z, Mir PS (1999). Localization and activity of rRNA genes on fenugreek (*Trigonella foenum-graecum* L.) chromosomes by fluorescent *in situ* hybridization and silver staining. Theoret. Appl. Genetics.

[26] Wicke S, Costa A, Muñoz J, Quandt D (2011). Restless 5S: The re-arrangement(s) and evolution of the nuclear ribosomal DNA in land plants. Mol. Phylogenet. Evol..

[27] Richards EJ, Ausubel FM (1988). Isolation of a higher eukaryotic telomere from *Arabidopsis thaliana*. Cell.

[28] Novák P, Neumann P, Pech J, Steinhaisl J, Macas J (2013). RepeatExplorer: a Galaxy-based web server for genome-wide characterization of eukaryotic repetitive elements from next-generation sequence reads. Bioinformatics.

[29] Novák P (2017). TAREAN: a computational tool for identification and characterization of satellite DNA from unassembled short reads. Nucleic Acids Res..

[30] Waminal NE (2017). A refined *Panax ginseng* karyotype based on an ultra-high copy 167-bp tandem repeat and ribosomal DNAs. J. Ginseng Res..

[31] Waminal NE, Pellerin RJ, Jang W, Kim HH, Yang T-J (2018). Characterization of chromosome-specific microsatellite repeats and telomere repeats based on low coverage whole genome sequence reads in *Panax ginseng*. Plant Breed. Biotech..

[32] Bradley, S., Zamechek, L. & Aurich-Costa, J. In *Fluorescence in Situ Hybridization (FISH)—Application Guide* (ed. T. Liehr) 67–73 (2009).

[33] Amann R, Fuchs BM (2008). Single-cell identification in microbial communities by improved fluorescence *in situ* hybridization techniques. Nat. Rev. Microbiol..

[34] Jensen MA, Webster JA, Straus N (1993). Rapid identification of bacteria on the basis of polymerase chain reaction-amplified ribosomal DNA spacer polymorphisms. Appl. Environ. Microbiol..

[35] Han, Y., Zhang, T., Thammapichai, P. & Weng, Y. Chromosome-specific painting in *cucumis* species using bulked oligonucleotides. *Genetics* (2015). 10.1534/genetics.115.177642 PMC4512542 25971668

[36] Fu S (2015). Oligonucleotide probes for ND-FISH analysis to identify rye and wheat chromosomes. Sci. Rep..

[37] Tang Z, Yang Z, Fu S (2014). Oligonucleotides replacing the roles of repetitive sequences pAs1, pSc119.2, pTa-535, pTa71, CCS1, and pAWRC.1 for FISH analysis. J. Appl. Genet..

[38] Kato A, Lamb JC, Birchler JA (2004). Chromosome painting using repetitive DNA sequences as probes for somatic chromosome identification in maize. PNAS.

[39] Sýkorová E (2003). Telomere variability in the monocotyledonous plant order Asparagales. Proc. R. Soc. Lond., B, Biol. Sci..

[40] Peška V (2015). Characterisation of an unusual telomere motif (TTTTTTAGGG)_n_ in the plant *Cestrum elegans* (Solanaceae), a species with a large genome. Plant J..

[41] Galian JA, Rosato M, Rossello JA (2012). Early evolutionary colocalization of the nuclear ribosomal 5S and 45S gene families in seed plants: evidence from the living fossil gymnosperm *Ginkgo biloba*. Heredity.

[42] Garcia S (2009). Linkage of 35S and 5S rRNA genes in *Artemisia* (family Asteraceae): first evidence from angiosperms. Chromosoma.

[43] Garcia S, Kovarik A (2013). Dancing together and separate again: gymnosperms exhibit frequent changes of fundamental 5S and 35S rRNA gene (rDNA) organisation. Heredity.

[44] Govaerts R, xeb. (2001). How many species of seed plants are there?. Taxon.

[45] Vitales D, D’Ambrosio U, Gálvez F, Kovařík A, Garcia S (2017). Third release of the plant rDNA database with updated content and information on telomere composition and sequenced plant genomes. Plant Syst. Evol..

[46] Son J-H (2012). Sequence variation and comparison of the 5S rRNA sequences in *Allium* species and their chromosomal distribution in four *Allium* species. J. Plant. Biol..

[47] Szymanski M, Barciszewska MZ, Erdmann VA, Barciszewski J (2002). 5S Ribosomal RNA Database. Nucleic Acids Res..

[48] Yevshin I, Sharipov R, Valeev T, Kel A, Kolpakov F (2017). GTRD: a database of transcription factor binding sites identified by ChIP-seq experiments. Nucleic Acids Res..

