# Supplementary material for: Rapid and Efficient FISH using Pre-Labeled Oligomer Probes
Source: Sci Rep. 2018 May 29;8:8224. doi: 10.1038/s41598-018-26667-z (PMC5974128; doi:10.1038/s41598-018-26667-z)
Supplement: Supplementary file 1 — Supplementary Information [file 41598_2018_26667_MOESM1_ESM.docx]

**Supplementary Information**

**Rapid and Efficient FISH using Pre-Labeled Oligomer Probes**

Nomar Espinosa Waminal^1,3^, Remnyl Joyce Pellerin^1^, Nam-Soo Kim^2^, Murukarthick Jayakodi^3^, Jee Young Park^3^, Tae-Jin Yang^3^*, and Hyun Hee Kim^1^*

^1^Chromosome Research Institute, Department of Life Science, Sahmyook University, Seoul, 01795, Korea

^2^Department of Molecular Biosciences, Kangwon National University, Chuncheon, **24341**, Korea

^3^Department of Plant Science, Plant Genomics and Breeding Institute, and Research Institute of Agriculture and Life Sciences, College of Agriculture and Life Sciences, Seoul National University, Seoul, 08826, Korea

*Corresponding authors: Correspondence and requests for materials should be addressed to T.J.Y. (tjyang@snu.ac.kr) or H.H.K (kimhh@syu.ac.kr)


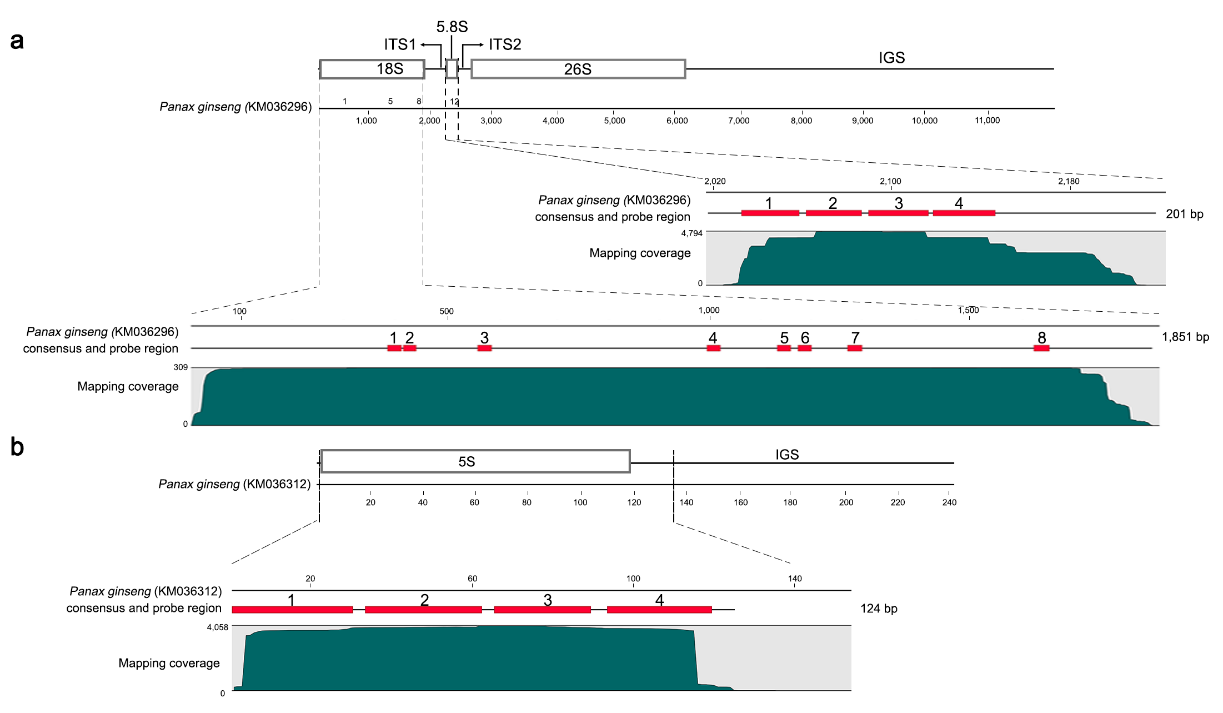
**Supplementary Figures**

**Supplementary Fig. S1. Construction of rDNA consensus sequences.**

**(a**) Mapping of 18S and 5.8S rDNA sequences from NCBI to the complete *Panax ginseng* 45S rDNA unit (KM036296) reference sequence generated 1,851-bp and 201-bp consensus sequences, respectively, from which PLOPs (red bars) were designed. (**b**) Mapping of 5S rDNA sequences from NCBI to the complete *Panax ginseng* 5S rDNA unit (KM036312) reference sequence generated a 124-bp consensus sequence from which PLOPs (red bars) were designed.


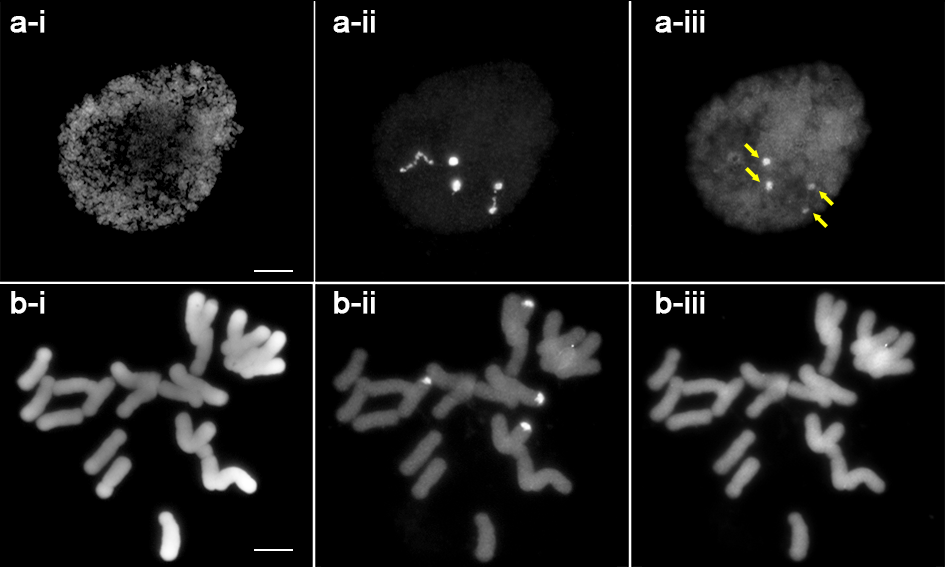


**Supplementary Fig. S2. FISH signals from 45S and 5S rDNA PLOPs in *G. biloba*.**

(**a**) Interphase and (**b**) metaphase chromosomes. Panels i–iii show raw images for DAPI, 45S, and 5S rDNA signals, respectively. An interphase cell in a-iii shows 5S rDNA signals at the 45S rDNA loci (arrows), which were not observed in metaphase chromosomes (b-iii). Bars = 10 μm.


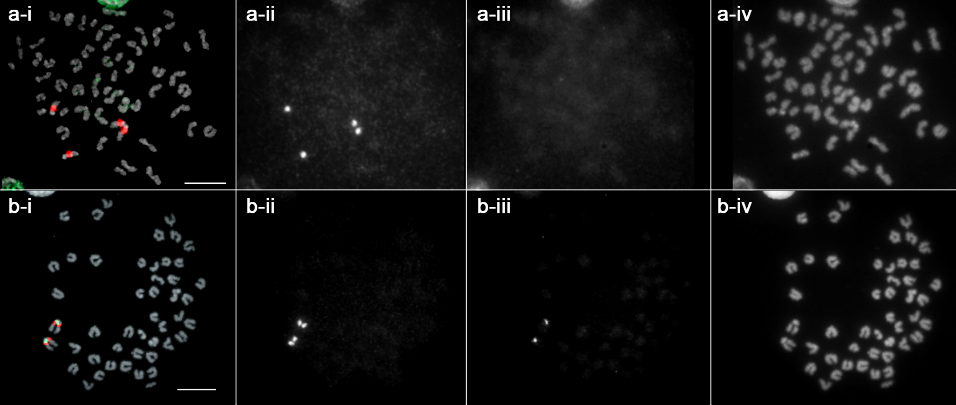

**Supplementary Fig. S3. FISH using PLOP cocktail on metaphase chromosomes of *Paralichthys olivaceus.***

(**a**) FISH with a pool of 12 PLOPs from 45S rDNA, four PLOPs from angiosperm-derived 5S rDNA, and *Arabidopsis*-type telomere probe cocktail showing signals from only the 45S rDNA probe. (**b**) FISH using a new set of 5S rDNA probes optimized for cranial vertebrates revealed clear 5S rDNA signals (b-iii). Panels i–iv show merged signals, raw 45S rDNA, raw 5S rDNA, and raw telomeric PLOP signals, respectively. Note that *P. olivaceus* did not show *Arabidopsis*-type telomeric signals; see Discussion. Bars = 10 μm.


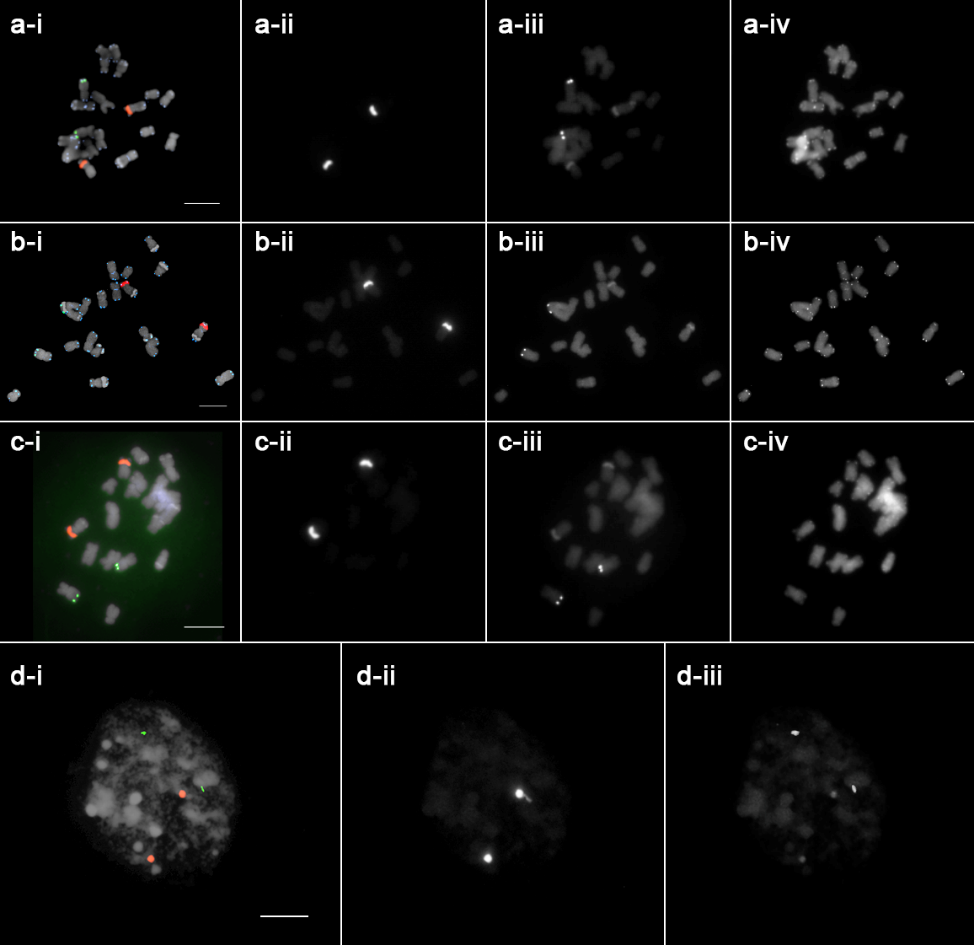


**Supplementary Fig. S4. FISH signals from the 5S, 45S, and telomeric PLOPs on *Zea mays* chromosomes**

FISH hybridization was carried out for (**a**) 5 min, (**b**) 1 hr, and (**c**) 7 hrs to test the efficiency of different durations of PLOP hybridization using metaphase chromosomes. (**d**) FISH signals from 45S and 5S rDNA on interphase cells. Panels i–iv show merged, raw 45S rDNA, raw 5S rDNA, and raw telomeric PLOP signals, respectively. Bars = 10 μm.


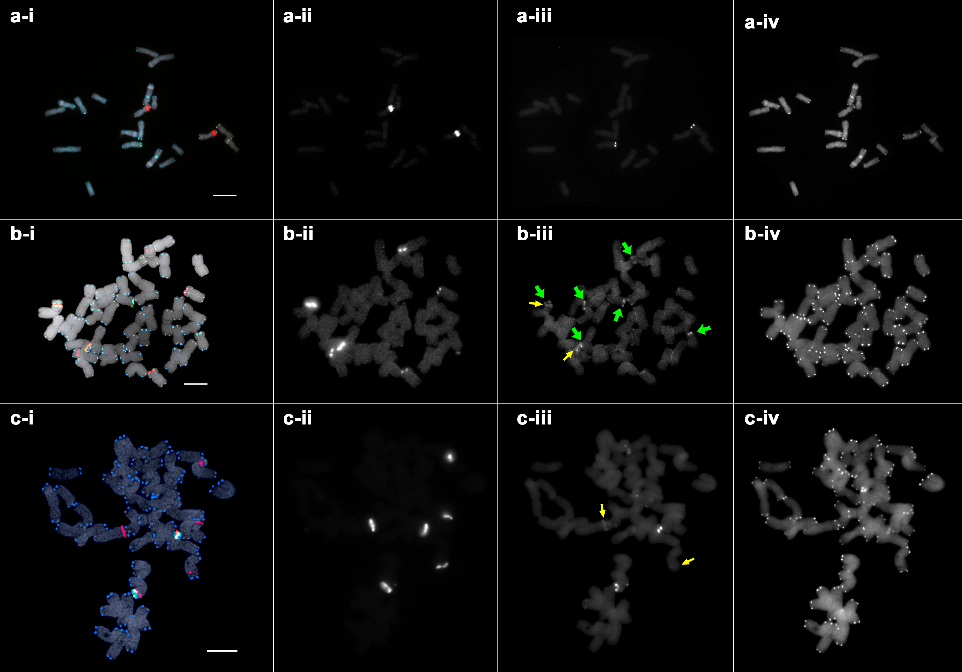


**Supplementary Fig. S5. PLOP-FISH signals in three monocot angiosperm species.**

(**a**) *Zea mays*, (**b**) *Triticum aestivum*, and (**c**) x*Triticosecale*. Panels i–iv show merged, raw 45S rDNA, raw 5S rDNA, and raw telomeric PLOP signals, respectively. Green and yellow arrows indicate weak 5S rDNA signals and 45S signal crosstalk in the FITC filter, respectively. Bars = 10 μm.


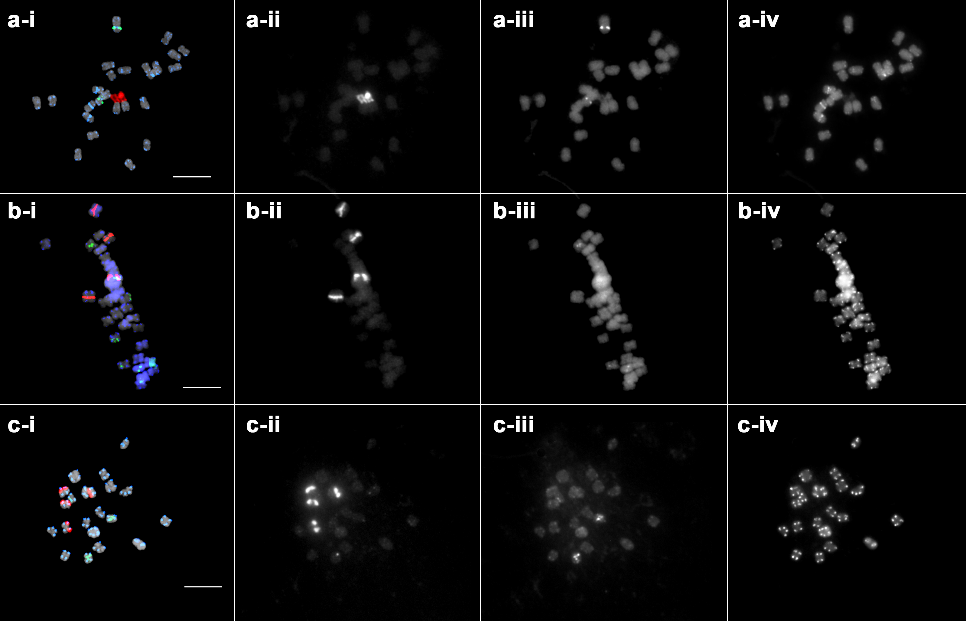


**Supplementary Fig. S6. PLOP-FISH signals in three dicot angiosperm species.**

(**a**) *Solanum commersonii,* (**b**) *Medicago sativa,* and (**c**) *Vigna angularis*. Panels i–iv show merged, raw 45S rDNA, raw 5S rDNA, and raw telomeric PLOP signals, respectively. Bars = 10 μm.


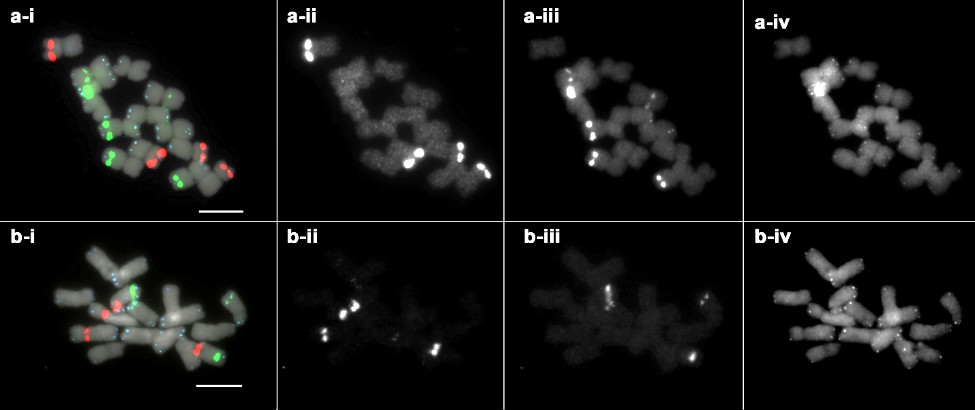


**Supplementary Fig. S7. PLOP-FISH signals using four PLOPs for each 45S and 5S rDNA.**

(**a**) *Hordeum vulgare* and (**b**) *Trigonella foenum-graecum*. Panels i–iv show merged, raw 45S rDNA, raw 5S rDNA, and raw telomeric PLOP signals, respectively. There is no observed Cy3 45S rDNA signal crosstalk with 5S rDNA FITC filter (See text). Bars = 10 μm.


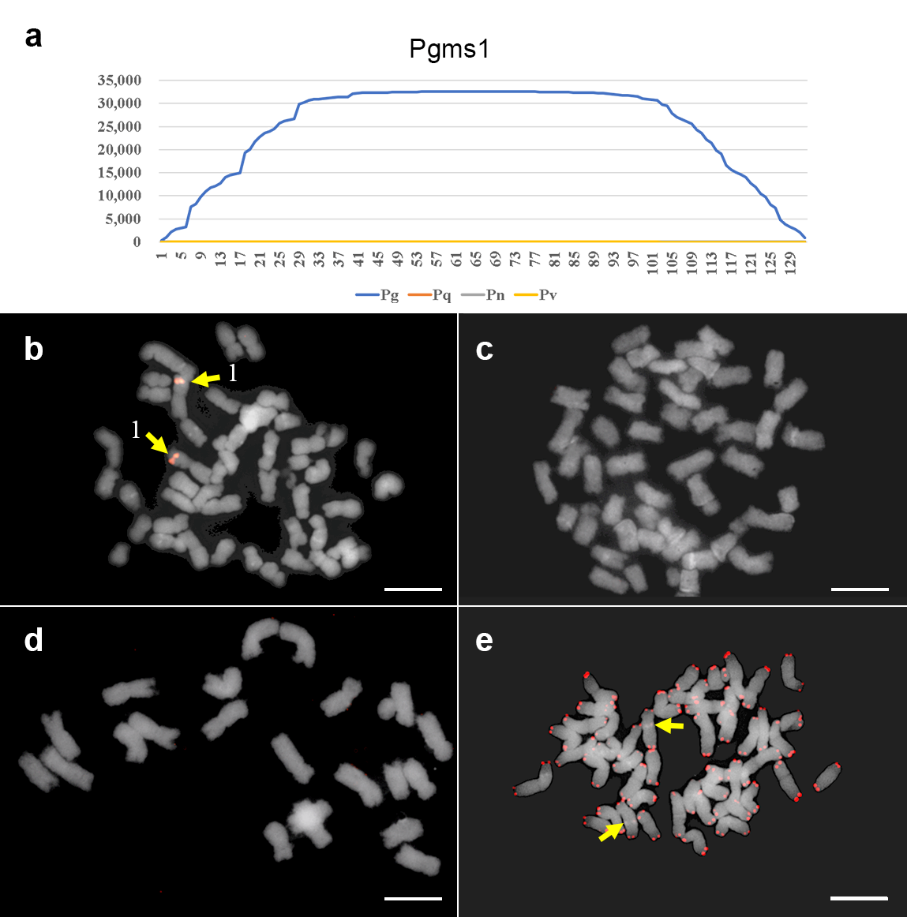


**Supplementary Fig. S8. An application of PLOP-FISH to detect newly identified specific tandem repeats in *P. ginseng*.** (**a**) Pgms1 was identified in *P. ginseng* and comparative WGS read mapping of related *Panax* species revealed its specific abundance in *P. ginseng*. Pg = *P. ginseng*, Pq = *P. quinquefolius*, Pn = *P. notoginseng*, Pv = *P. vietnamensis*. (**b–d**) PLOP-FISH analysis of Pgms1 hybridized to *P. ginseng* (yellow arrows), *P. quinquefolius*, and *P. notoginseng* chromosomes, respectively, supports data from **a**. (**e**) FISH signals from the *Arabidopsis-*type telomere sequences (TTTAGGG)_n_ in *P. ginseng* chromosomes. Yellow arrows indicate interstitial sites of telomeric repeats. Bars = 10 μm.

**Supplemmentary Tables**

**(S1, S2, S6, and S7. For S3~S5, see attached excel dataset)**

# Supplementary Table S1. Mapping summary of rDNA sequences from NCBI

|  | | | | | |
| --- | --- | --- | --- | --- | --- |
| **rDNA type** | **Reference** | **Total sequences** | **Mapped sequences** | **(%)** |  |
| 18S | KM036296 | 414 | 314 | 76 |  |
| 5.8S | KM036296 | 7,249 | 4,794 | 66 |  |
| 5S | KM036312 | 6,750 | 4,058 | 60 |  |

# Supplementary Table S2. Consensus sequences obtained after reference mapping of rDNA sequences

| **rDNA family** | **Consensus sequence** | **Length (bp)** |
| --- | --- | --- |
| 18S rDNA | TACCTGGTTGATCCTGCCAGTAGTCATATGCTTGTCTCAAAGATTAAGCCATGCATGTGTAAGTATGAACTAATTCAGACTGTGAAACTGCGAATGGCTCATTAAATCAGTTATAGTTTGTTTGATGGTATCTGCTACTCGGATAACCGTAGTAATTCTAGAGCTAATACGTGCAACAAACCCCGACTTCTGGAAGGGATGCATTTATTAGATAAAAGGTCGACGCGGGCTTCTGCCCGTTGCTCTGATGATTCATGATAACTCGACGGATCGCACGGCCTTCGTGCCGGCGACGCATCATTCAAATTTCTGCCCTATCAACTTTCGATGGTAGGATAGTGGCCTACTATGGTGGTGACGGGTGACGGAGAATTAGGGTTCGATTCCGGAGAGGGAGCCTGAGAAACGGCTACCACATCCAAGGAAGGCAGCAGGCGCGCAAATTACCCAATCCTGACACGGGGAGGTAGTGACAATAAATAACAATACCGGGCTCTTCGAGTCTGGTAATTGGAATGAGTACAATCTAAATCCCTTAACGAGGATCCATTGGAGGGCAAGTCTGGTGCCAGCAGCCGCGGTAATTCCAGCTCCAATAGCGTATATTTAAGTTGTTGCAGTTAAAAAGCTCGTAGTTGGACCTTGGGTTGGGTCGACCGGTCCGCCTCTCGGTGTGCACCGGTCGTCTCGTCCCTTCTGCCGGCGATGCGCTCCTGGCCTTAATTGGCCGGGTCGTGCCTCCGGCGCTGTTACTTTGAAGAAATTAGAGTGCTCAAAGCAAGCCTACGCTCTGGATACATTAGCATGGGATAACATCATAGGATTTCGGTCCTATTGTGTTGGCCTTCGGGATCGGAGTAATGATTAACAGGGACAGTCGGGGGCATTCGTATTTCATAGTCAGAGGTGAAATTCTTGGATTTATGAAAGACGAACAACTGCGAAAGCATTTGCCAAGGATGTTTTCATTAATCAAGAACGAAAGTTGGGGGCTCGAAGACGATCAGATACCGTCCTAGTCTCAACCATAAACGATGCCGACCAGGGATCGGCGGATGTTGCTTTTAGGACTCCGCCGGCACCTTATGAGAAATCAAAGTTTTTGGGTTCCGGGGGGAGTATGGTCGCAAGGCTGAAACTTAAAGGAATTGACGGAAGGGCACCACCAGGAGTGGAGCCTGCGGCTTAATTTGACTCAACACGGGGAAACTTACCAGGTCCAGACATAGTAAGGATTGACAGACTGAGAGCTCTTTCTTGATTCTATGGGTGGTGGTGCATGGCCGTTCTTAGTTGGTGGAGCGATTTGTCTGGTTAATTCCGTTAACGAACGAGACCTCAGCCTGCTAACTAGCTATGCGGAGGTAACCCTCCGCGGCCAGCTTCTTAGAGGGACTATGGCCGTTTAGGCCAAGGAAGTTTGAGGCAATAACAGGTCTGTGATGCCCTTAGATGTTCTGGGCCGCACGCGCGCTACACTGATGTATTCAACGAGTCTATAGCCTTGGCCGACAGGCCCGGGTAATCTTTGAAATTTCATCGTGATGGGGATAGATCATTGCAATTGTTGGTCTTCAACGAGGAATTCCTAGTAAGCGCGAGTCATCAGCTCGCGTTGACTACGTCCCTGCCCTTTGTACACACCGCCCGTCGCTCCTACCGATTGAATGGTCCGGTGAAGTGTTCGGATCGCGGCGACGTGGGCGGTTCGCTGCCGGCGACGTCGCGAGAAGTCCACTGAACCTTATCATTTAGAGGAAGGAGAAGTCGTAACAAGGTTTCCGTAGGTGAACCTGCGGAAGGATCATTGTCGAAACCTGCACAGCAGAACGACCCGCGAACACGTTACAA | 1,851 |
| 5.8S rDNA | TCTTTCTAAAACACAAACGACTCTCGGCAACGGATATCTCGGCTCTCGCATCGATGAAGAACGTAGCGAAATGCGATACTTGGTGTGAATTGCAGAATCCCGTGAACCATCGAGTCTTTGAACGCAAGTTGCGCCCGAAGCCATTAGGCCGAGGGCACGTCTGCCTGGGCGTCACGCATCGCGTCGCCCCCCAACCCATCA | 201 |
| 5S rDNA (angiosperm) | GGGTGCGATCATACCAGCACTAAAGCACCGGATCCCATCAGAACTCCGAAGTTAAGCGTGCTTGGGCGAGAGTAGTACTAGGATGGGTGACCTCCTGGGAAGTCCTCGTGTTGCACCCCTTTTT | 124 |
| 5S rDNA (gymnosperm) | GGGTGCGATCATACCAGCGTTAATGCACCGGATCCCATCAGAACTCCGCAGTTAAGCGCGCTTGGGCTAGAGTAGTACTGGGATGGGTGACCTCCCGGGAAGTCCTAGTGTTGCACCCTC | 120 |
| 5S rDNA (cranial vertebrate) | GCCTACGGCCATACCACCCTGAACGCGCCCGATCTCGTCTGATCTCGGAAGCTAAGCAGGGTCGGGCCTGGTTAGTACTTGGATGGGAGACCGCCTGGGAATACCGGGTGCTGTAGGCT | 119 |

# Supplementary Table S6. List of species used and distribution of PLOPs

| **Scientific name** | **Common name** | **Group** | **Chr. no. (2n)** | **45S rDNA count** | **5S rDNA count** | **Telomere location** | **Source** |
| --- | --- | --- | --- | --- | --- | --- | --- |
| *Medicago sativa* subps*. sativa* | Alfalfa | Angiosperm - dicot | 32 | 4 | 4 | terminal | National Agrobiodiversity Center, RDA |
| *Phaseolus vulgaris* var*. vulgaris* | Common bean | Angiosperm - dicot | 22 | 8 | 4 | terminal | National Agrobiodiversity Center, RDA |
| *Solanum commersonii* | Wild potato | Angiosperm - dicot | 24 | 2 | 2 | intercalary, terminal | Highland Agriculture Research Institute, NICS, RDA |
| *Trigonella foenum-graecum* | Fenugreek | Angiosperm - dicot | 16 | 8 | 4 | terminal | National Agrobiodiversity Center, RDA |
| *Vigna angularis* var*. angularis* | Adzuki bean | Angiosperm - dicot | 22 | 4 | 2 | terminal | National Agrobiodiversity Center, RDA |
| *Allium cepa* var*. cepa* | Onion | Angiosperm - monocot | 16 | 2 | 2 | -- | National Agrobiodiversity Center, RDA |
| *Hordeum vulgare* subps*. vulgare* | Barley | Angiosperm - monocot | 14 | 6 | 8 | terminal | National Agrobiodiversity Center, RDA |
| *Triticum aestivum* subps*. aestivum* | Bread wheat | Angiosperm - monocot | 42 | 6 | 8 | terminal | National Agrobiodiversity Center, RDA |
| x*Triticosecale* sp. | Hybrid wheat and rye | Angiosperm - monocot | 42 | 6 | 8 | terminal | National Agrobiodiversity Center, RDA |
| *Zea mays* | Maize | Angiosperm - monocot | 20 | 2 | 2 | terminal | Seoul Women's University |
| *Paralichthys olivaceus* | Olive flounder | Animalia - fish | 48 | 2 | 2 | -- | National Institute of Fishiries Science |
| *Ginkgo biloba* | Ginkgo | Gymnosperm | 24 | 4* | 4 | terminal | Department of Herbal Crop Research, NIHHS, RDA |
| *Pinus densiflora* | Pine | Gymnosperm | 24 | 14 | 2 | intercalary, terminal | National Forest Seed Variety Center |
| *All signals colocalized with 5S rDNA | | | | | | | |
|  | | | | | | | |

# Supplementary Table S7. Summary of WGS reads used for comparative WGS mapping of Pgms1 among *Panax ginseng*-related species

| **Species** | **2*n*** | **Ploidy** | **Genome size (Mb)** | **WGS size (Mbp)** | **x^a^** |
| --- | --- | --- | --- | --- | --- |
| *P. ginseng* (Chunpoong) | 48 | 4x | 3,600 | 2,662 | 0.74 |
| *P. quinquefolius* | 48 | 4x | 4,914 | 1,183 | 0.24 |
| *P. notoginseng* | 24 | 2x | 2,454 | 1,989 | 0.81 |
| *P. vietnamensis* | 24 | 2x | 2,018 | 3,182 | 1.58 |

^a^ Equivalent genome coverage of extracted WGS reads used for analysis
